# Amides as Non-polymerizable Catalytic Adjuncts Enable the Ring-Opening Polymerization of Lactide With Ferrous Acetate Under Mild Conditions

**DOI:** 10.3389/fchem.2019.00346

**Published:** 2019-05-16

**Authors:** Toufik Naolou, Andreas Lendlein, Axel T. Neffe

**Affiliations:** ^1^Institute of Biomaterial Science, Helmholtz-Zentrum Geesthacht, Teltow, Germany; ^2^Institute of Chemistry, University of Potsdam, Potsdam, Germany

**Keywords:** ring-opening polymerization, polyester, catalyst, iron, amide ligand

## Abstract

Sn-based catalysts are effective in the ring-opening polymerization (ROP) but are toxic. Fe(OAc)_2_ used as an alternative catalyst is suitable for the ROP of lactide only at higher temperatures (>170°C), associated with racemization. In the ROP of ester and amide group containing morpholinediones with Fe(OAc)_2_ to polydepsipeptides at 135°C, ester bonds were selectively opened. Here, it was hypothesized that ROP of lactones is possible with Fe(OAc)_2_ when amides are present in the reactions mixture as Fe-ligands could increase the solubility and activity of the metal catalytic center. The ROP of lactide in the melt with Fe(OAc)_2_ is possible at temperatures as low as 105°C, in the presence of *N*-ethylacetamide or *N*-methylbenzamide as non-polymerizable catalytic adjuncts (NPCA), with high conversion (up to 99 mol%) and yield (up to 88 mol%). Polydispersities of polylactide decreased with decreasing reaction temperature to ≤ 1.1. NMR as well as polarimetric studies showed that no racemization occurred at reaction temperatures ≤145°C. A kinetic study demonstrated a living chain-growth mechanism. MALDI analysis revealed that no side reactions (e.g., cyclization) occurred, though transesterification took place.

## Introduction

**Figure 1:**
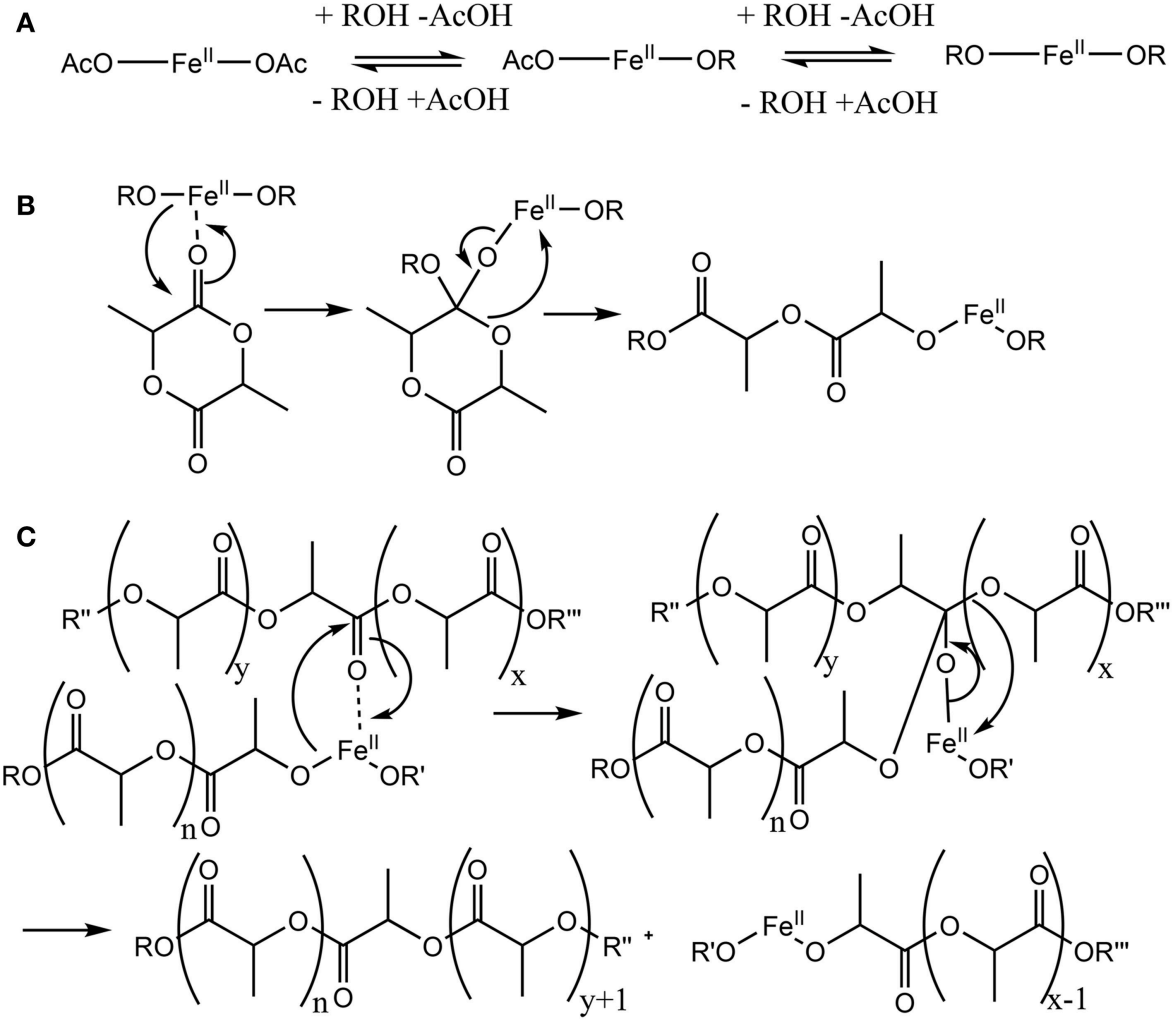
Putative mechanism of the Fe(II)-catalyzed ring-opening polymerization (ROP) of dilactide by coordination and insertion. **(A)** An exchange of acylate and alkoxide ligands of the catalytically active metal center precedes the actual ROP. **(B)** In the catalytic cycle, a monomer coordinates to the catalytic active metal, followed by a transfer of an alkoxide ligand to form a tetrahedral intermediate. Subsequently, the ring opening leads to the reformation of an ester bond and the elongation of the growing chain by two lactic acid units coordinated to iron by the terminal alkoxide group. **(C)** As side reaction, often increasingly observed in ROP at high reaction temperatures and/or monomer conversion, transesterification may take place which result in species with 2n+1 lactic acid units rather than with 2n lactic acid units, which would be expected when only the ring-opening takes place.

(Co)polyesters obtained from diglycolide, dilactide, or ε-caprolactone are typical representatives of hydrolytically and enzymatically degradable polymers that are nowadays employed e.g., as matrix for drug delivery systems (Wischke and Schwendeman, 2008; Kumari et al., 2010; Hu et al., 2013) as well as for some temporary implants (Grafahrend et al., 2011; Zhang et al., 2011). The molecular structure and the material properties of (co)polyesters, e.g., their molar mass, crystallinity, hydrophobicity, and tacticity, play essential roles for their functional capabilities, such as the rate of degradation, structural function or drug release rates (Neffe et al., 2010). While several synthetic routes to (co)polyesters have been developed, ring-opening polymerization (ROP) of cyclic precursor lactones has many advantages, such as gaining higher molar mass and lower polydispersity than is accessible by polycondensation. The ability to control the end groups of the resulting polymer chains provides a versatile method to prepare telechelics. ROP of lactones can be conducted under anionic or cationic conditions, by Lewis-acidic organometallic catalysts, (Jérôme and Lecomte, 2008) organocatalysts, (Kamber et al., 2007), and enzymes (Matsumura et al., 1997; Numata et al., 2007). Organometallic catalysts have been demonstrated to be highly efficient in tailoring the molecular weight of polyesters. These catalysts typically follow a coordination-insertion mechanism starting from a metal alkoxide added as a catalyst (e.g., aluminiumalkoxides) or formed *in situ* [e.g., tin(II)octoate, compare also (Figure 1)]. While these two catalysts are the ones predominantly used, literally hundreds of catalysts are known (Dechy-Cabaret et al., 2004; Wheaton et al., 2009; Ajellal et al., 2010). Two potential drawbacks associated with most of these catalysts are the potential toxicity of the catalysts, (Tanzi et al., 1994; Egorova and Ananikov, 2016), which need to be carefully removed for biomedical use of the polymer, (Xiao et al., 2012) and the availability of the catalysts, which for more elaborated catalysts comprises multi-step synthesis under water-free conditions. Further important selection criteria for a suitable catalyst are the required activation energy/reaction temperature, as high temperatures promote racemization, the solubility of the catalyst in the monomer melt, or, less frequently used, in a suitable solvent.

Fe(II) and Fe(III) compounds have been proposed as a less toxic alternative to Sn-based catalysts in the ROP of lactones, such as dilactide (Gibson et al., 2002; Wang et al., 2005; Chen et al., 2006; Hege and Schiller, 2014; Geng et al., 2016). The toxicity of tin compounds is related to disturbance of the iron and copper metabolism, as well as the potential denaturation of proteins by reaction with free thiols (Westrum and Thomassen, 2002; Buck et al., 2003). Though Fe(II) as well as Fe(III) ions, in high concentrations, may have toxic effects, typically through redox reactions forming radicals, biological organisms have developed complexing strategies that typically allow safe transport and storage of iron ions within the organisms (Imlay and Linn, 1988; Stohs and Bagchi, 1995). Figure 1 shows the pre-equilibrium between metal carboxylate and alkoxide (Figure 1A) (Zhang et al., 1994; Kowalski et al., 2005), as well as the coordination-insertion mechanism of the ROP (Figure 1B) in analogy to the mechanism of Sn(Oct)_2_, demonstrating that this reaction, in the case of dilactide, will add exactly two monomers during the addition of each lactone. Furthermore, the catalysts may promote transesterification of oligomers and polymers (Figure 1C), which is typically observed at high temperatures and/or high conversions. Such transesterification would lead to oligomers and polymers of the structure Initiator-(LA)_x_ rather than Initiator-(LA)_2x_, and is desired when statistical copolymers are targeted.

**Figure 2:**
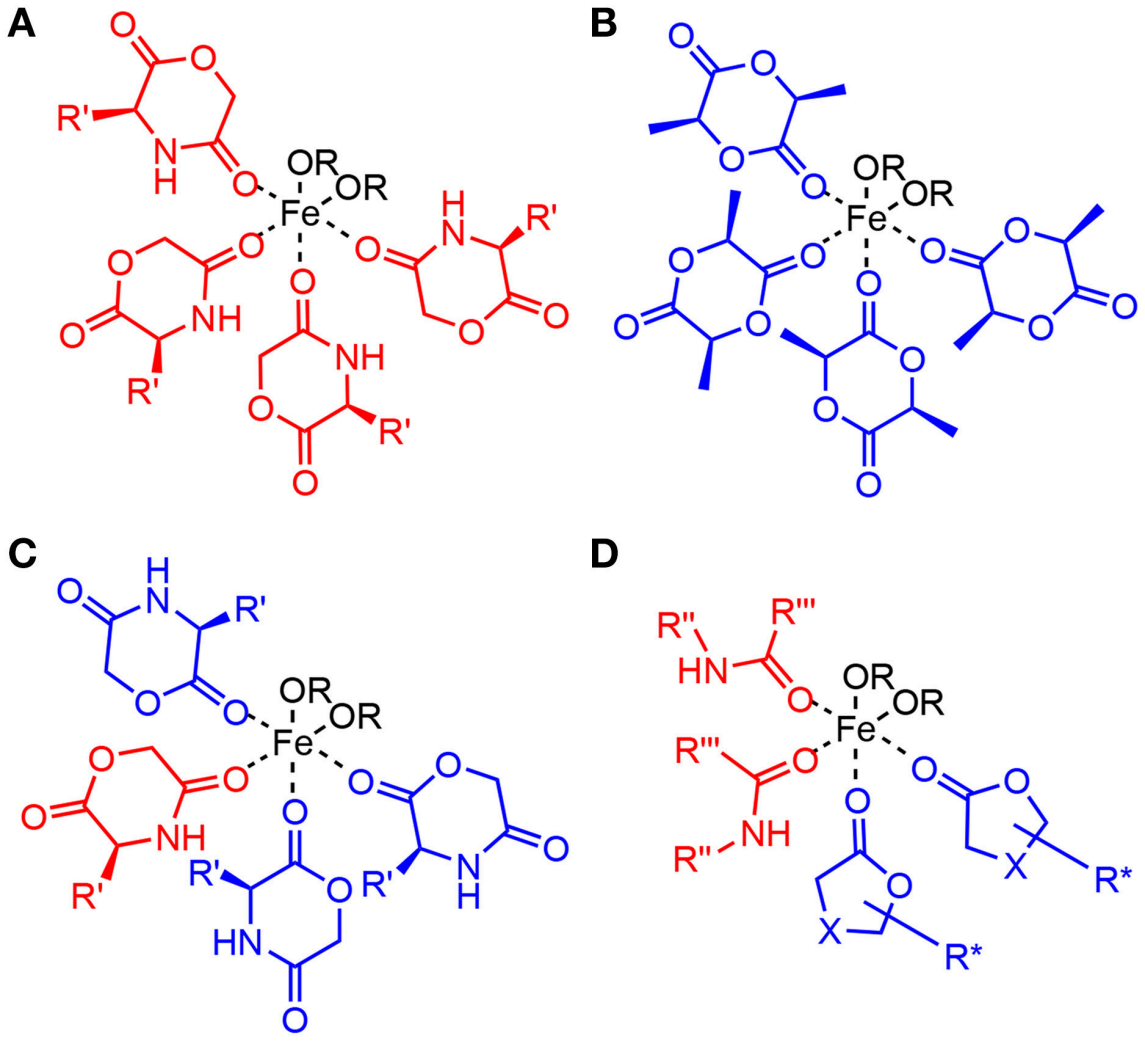
Models of potential octahedric coordination of Fe(II) in the ring-opening polymerization of lactones. In addition to two alkoxide ligands **(A)** shows four morpholinediones coordinated via the amide group. Such coordination has been reported, but is inconsistent with the observed selective reaction of the ester bond. **(B)** contains four dilactide units, as expected in the ROP of lactide without NPCA, but the ROP of dilactide by Fe(OAc)_2_ is ineffective. **(C)** ROP of morpholinediones likely proceeds via a complex with mixed amide and ester coordination. **(D)** depicts the proposed active center for the ROP of lactones by Fe(II) in the presence of NPCAs, which also has such mixed coordination.

While this general mechanism is well-established in polymer chemistry, it is simplified, as Fe(II), and many other typical catalytic metals used for this reaction, preferentially adopt a octahedral coordination rather than two- or three ligands as shown in the generic mechanism. In the solid state, Fe(OAc)_2_ crystallizes in a 3D network being bridged by multidentate acetate ligands adopting four different types of coordination (Weber et al., 2011). For the melt, the exact coordination is not known, though modeling studies for Sn(Oct)_2_ suggest a coordination of alcohols and carboxylic acids, as well as monomers to the central metal (Ryner et al., 2001). We recently showed that in the case of morpholinediones, Fe(OAc)_2_ was effective in the ROP by selectively opening the ester bond, while the amide bond was preserved (Naolou et al., 2016). However, Fe(OAc)_2_ for ROP of dilactide was judged to be unreactive (Kricheldorf et al., 2000), or could only be used at high temperatures promoting racemization (Stolt and Södergård, 1999). These somewhat contradictory results may suggest that while amide coordination to Fe(II) occurs (Ding et al., 2009), selective coordination of the morpholinedione to Fe(II) via the amide bond (Figure 2A) leads to an unreactive species. On the other hand, systems without the amide functionality, such as that represented in Figure 2B, do not show any activity. Hence, it is likely that the catalytically active Fe(II) is octahedrically coordinated to different types of ligands, i.e., (i) alkoxide ligands, which are the initiator or the growing chain, (ii) monomers coordinated via the ester group, which are transferred to the growing chain, and (iii) amides, stabilizing and activating the metallic center (Figure 2C).

We therefore hypothesized an Fe(II) catalyst active in the ROP of lactones such as L,L-dilactide can be formed from Fe(OAc)_2_ *in situ* by adding the lactone to be polymerized and an initiator, and in addition non-polymerizable amides as catalyst adjuncts (NPCA) to the reaction mixture. The role of the NPCA could be to allow this type of coordination to increase the solubility in the melt or to change the stereoelectronic properties of the catalyst so that the coordination and insertion is promoted. The latter requires a fine balance between Lewis acidity and softness of the catalyst as well as minimal steric hinderance so that coordination, insertion, and chain transfer are occurring effectively. By adding the adjuncts, the active complex would form spontaneously and would not require synthesis and purification (Figure 2D).

Our concept for testing the hypothesis was to study the ROP of dilactide with Fe(OAc)_2_ in the presence (or absence) of *N*-ethylacetamide (NEAA) or *N*-methylbenzamide (NMBA) as NPCAs. These two adjuncts can be removed during the precipitation step of the synthesis as they are soluble in methanol used for the precipitation. By conducting the reaction at different temperatures, investigating monomer conversion at different time points, polymer yield, molar mass, polymer composition and polydispersity as well as thermal transitions, and optical purity as material characteristics, the validity of the approach to add NPCAs was comprehensively tested. The kinetics of the reaction is described and the limits of the catalyst system, in terms of required temperatures and observed side reactions such as racemization and transesterification, were explored.

## Experimental Section

### Materials

L,L-dilactide was purchased from Corbion (Gorinchem, The Netherlands) and purified by recrystallization from anhydrous toluene. 1,8-Octanediol 98%, 1-Methyl-2-pyrrolidinone (anhydrous) 99.5% (NMP), iron(II) acetate (Fe(OAc)_2_) ≥99.99%, *N*-ethylacetamide 99%, *N*-methylbenzamide ≥99%, methanol, and toluene (anhydrous) 99.8% were purchased from Sigma-Aldrich (Schnelldorf, Germany) and used as received. Chloroform 99% was obtained from Roth (Karlsruhe, Germany), tetrahydrofuran (for liquid chromatography) from Merck (Darmstadt, Germany).

### Synthesis of Poly(L-lactide) Diol: (PLLA)

In a typical procedure to synthesize PLLA in bulk, 1.5 g (10.4 mmol) of L,L-dilactide, 14.6 mg (0.1 mmol, 1/104 eq) 1,8-Octanediol, and 11.2 mg (0.065 mmol, 1/160 eq) of Fe(OAc)_2_ were added to a 10 mL oven-dried Schlenk tube sealed with rubber septum. The solid mixture in the tube was shaken to get a uniform distribution for its components and were degassed by applying three vacuum/argon refill cycles. 0.16 ml (10 wt%) of *N*-ethylacetamide or 150 mg *N*-methylbenzamide was then added to the tube followed by a further vacuum/argon refill cycle. The tube was placed in a preheated oil bath for 4 h. The polymerization was stopped by adding 8 ml of chloroform to dissolve the resulting polymer, followed by precipitation in 500 ml of methanol. The polymer was collected and dried under vacuum at 60°C for 2 days to yield a white polymer, ^1^H NMR (500 MHz, CDCl_3_): δ = 5.33–4.86 (q, 217H, OC*H*CO), 4.37–4.22 (s br, 2H, COC*H*OH), 4.11–3.96 (m, 4H, 2 CH_2_C*H*_2_O), 2.03 – 1.15 (m, 680H, 222 CHC*H*_3_,2 C*H*_2_C*H*_2_C*H*_2_) ppm; ^13^C NMR (126 MHz, CDCl_3_): δ = 169.58 (CH*C*OO), 69.01 (O*C*HCO), 65.56 (O*C*H_2_CH_2_), 29.01(OCH_2_*C*H_2_), 28.41 (2 *C*H_2_CH_2_CH_2_CH_2_O), 25.63 (2 CH_2_*C*H_2_CH_2_CH_2_O), 16.65 (CH*C*H_3_) ppm.

A similar procedure was followed for the case of synthesis of PLLA using NMP as a solvent, except that the LLA and 1,8-Octanediol and Fe(OAc)_2_ were first dissolved in 2 ml of NMP, which was followed by a degassing process achieved by bubbling a stream of argon through the reaction solution for 20 min.

### Polymer Characterization

^1^H and ^13^C NMR spectra were recorded at room temperature using a DRX 500 Avance II spectrometer (500 MHz, Bruker, Rheinstetten, Germany; software Topspin version 1.3). Deuterated chloroform (CDCl_3_) was used as a solvent. The determination of the number average molecular weight M_n_ from the ^1^H spectra was performed by comparing the integrals of the C*H*_2_-O group of the initiator (4.11–3.96 ppm) with the C*H* protons of the lactic acid unit (5.33–4.86 and 4.37–4.22 ppm).

The GPC measurements were carried out using tetrahydrofuran or chloroform as an eluent at 35°C with a flow rate of 1 mL · min^−1^ and in the presence of 0.2 wt% toluene as the internal standard. The GPC system was equipped with a pre-column, two 300 mm × 8.0 mm linear M columns (Polymer Standards Service GmbH, Mainz, Germany, PSS), an isocratic pump 2080, and an automatic injector AS 2050 (both Jasco, Tokyo, Japan). Two detectors were used: a RI detector Shodex RI-101 (Showa Denko, Japan) and the viscosimeter SEC-3010 (WGE, Dr. Bures, Dallgow, Germany). Polymer molecular weights were evaluated using universal calibration obtained by applying polystyrene standards with M_n_ between 580 g · mol^−1^ and 975 000 g · mol^−1^ (PSS) using the SEC software WINGPC UniChrom V. 8.2.1 (PSS).

Differential scanning calorimetry (DSC) measurements were performed on a Netzsch DSC 204, Selb, Germany. The experiments were carried out under continuous nitrogen flow by heating a sample from room temperature up to 200 °C. The temperature was then kept at 200°C for 10 min followed by cooling down to −70°C, and again warming up to 200 °C with a constant heating and cooling rate of 10 K·min^−1^.

The specific rotation [α]_D_ of polymer solutions in chloroform was measured at a concentration of 5 mg·ml^−1^ and a temperature of 22°C, using a P-200 polarimeter (Jasco, Groß-Umstadt, Germany).

Mass spectra were measured on an ultrafleXtreme MALDI-ToF spectrometer (Bruker, Bremen, Germany). *trans*-2-[3-(4-*tert*-butylphenyl)-2-methyl-2-propenylidene]malononitrile (DCTB) was used as matrix and NaI to favor ionization by sodium attachment. PLLA (10 mg/mL in chloroform), the matrix (10 mg/mL in THF) and sodium ion source (10 mg/ml in THF) were mixed in a 1:10:1 volume ratio and then 1 μl pipetted on the matrix target for analysis.

All data presented in this manuscript are based on single synthesis experiments conducted by T.N. The reliability of the system has been proven by selected syntheses repeated by two additional experimenters, however, the data of these experiments have not been included here.

## Results and Discussion

**Figure 3:**
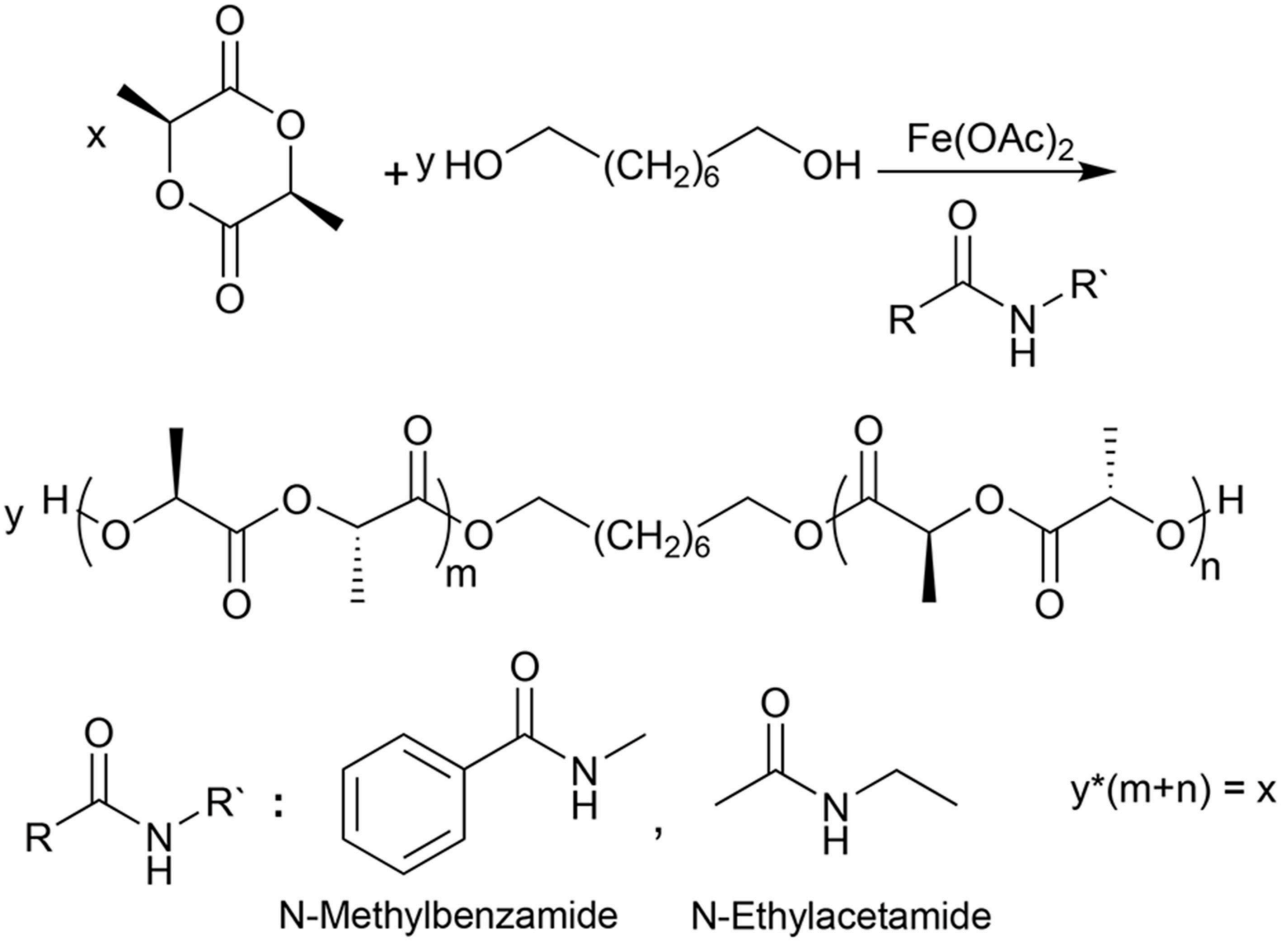
The synthetic route employed to prepare PLLA using 1,8-Octanediol as initiator, Fe(OAc)_2_ as a catalyst and *N*-ethylacetamide or *N*-methylbenzamide as NPCAs.

The ROP of L,L-dilactide with Fe(OAc)_2_ as a catalyst in the presence of *N*-ethylacetamide or *N*-methylbenzamide as NPCAs (Figure 3) was studied at 165, 145, 125, and 105°C, and the results are summarized in Table 1. The theoretical M_n_ to be synthesized at full conversion was in all cases 15 kDa, which was regulated by the ratio of 1,8-octandiol and L,L-dilactide. The yield of the respective reaction was determined by weight. The monomer conversion and molar mass were determined by ^1^H NMR spectroscopy. The molar mass and mass distribution of different PLLA samples were measured using gel permeation chromatography (GPC) equipped with two detectors employing universal calibration. The thermal transitions, change of heat capacity at T_g_, as well as the melting enthalpy of the PLLA samples were measured by differential scanning calorimetry (DSC), and the values from the 2nd heating run are reported. The specific rotations of the polymer solutions were determined by polarimetry.

**Table 1:** Results of the polymerization reactions.

	**no NPCA ^a^**		***N*****-ethylacetamide**				***N*****-methylbenzamide**			
T [°C]	165	145	165	145	125	105	165	145	125	105
Yield [wt%]	67	37	70	67	83	84	59	67	78	42
Conversion [mol%]	96	50	96	97	98	97	96	97	93	50
M_n, (NMR)_ [kDa]	21.6	11.2	16.5	18.5	17.3	16.7	15.6	16.8	15.6	10.0
M_n, (GPC)_ [kDa]	17.0	14.6	14.1	17.5	15.3	15.5	15.1	18	14.9	8.2
PDI	2	1.4	1.7	1.8	1.4	1.12	1.9	1.7	1.17	1.09
T_g_ [°*C*]^b^	54	51	53	51	53	51	52	51	53	50
Δ*C*_p_ [J·(*g*·*K*)^−1^]^b^	0.82	0.16	0.30	0.29	0.95	0.89	0.26	0.44	0.26	0.41
${\text{T}}_{\text{m}}^{1}$ [°*C*]^b^	154	147	149	152	155	153	148	149	152	151
$\Delta H_{\text{m}}^{1}$ [J·*g*^−1^]^b^	15	8	18	12	12	8	13	2	4	35
${\text{T}}_{\text{m}}^{2}$ [°*C*]^b^	162	159	155	161	164	163	158	164	164	156
$\Delta H_{\text{m}}^{2}$ [J·*g*^−1^]^b^	28	33	10	27	35	42	25	39	46	15
[α]^22^	−153	−155	−146	−151	−155	−153	−153	−156	−158	−143

*The reaction was performed for 4 h, with a catalyst/monomer ratio of 1:160, and with/without addition of 10 wt% NPCA (compared to the monomer). The theoretical M_n_ was 15 kDa*.

a At 125 or 105 °C, the polymerization did not take place without addition of an NPCA

b *2^nd^ heating run. Two T_m_s were observed*.

*Precision of methods: NMR: ~10%, GPC: < 10%, DSC: Enthalpy ~10%, Temperature: 1 K*.

The average number molecular weights of the polymers from all syntheses with yield ≥59% and conversion >50% was reasonably close to the theoretically expected M_n_. Without addition of a NPCA, an acceptable conversion and yield could only be reached at 165°C, while at 145°C conversion, when yield substantially dropped and at lower temperatures, the ROP was not successful. These results are in agreement with the literature (Stolt and Södergård, 1999; Kricheldorf et al., 2000), in which Fe(OAc)_2_ catalyzed the ROP of dilactides only at elevated temperatures. When Sn(Oct)_2_ is used, polymerizations are typically conducted above the melting temperature of PLLA (170–200°C). It has been shown that finalizing the polymerization at temperatures below the melting point of PLLA, a solid-phase polymerization occurs that leads to more complete conversions than can be reached in the melt (Shinno et al., 1997). Both *N*-ethylacetamide and *N*-methylbenzamide used as NPCA allowed for high conversions and yields, even at lower temperatures down to 105°C. At all times, the investigated reaction mixtures at this temperature, i.e., above the T_m_ of the L,L-dilactide monomer, behaved as clear solutions/melts. The polydispersity of the polymers decreased with the reaction temperature, and was lower than typically reported for Sn(Oct)_2_ (Degée et al., 1999), even when using solid-state polymerization (Shinno et al., 1997), which shows one benefit of the NPCA. The difference between conversion and yield correlated with the PDI, which can be interpreted by removal of smaller oligomers that are present in a larger fraction in samples with higher PDI than in samples with lower PDI, and have a higher solubility than larger oligomers and polymers during the precipitation step.

**Figure 4:**
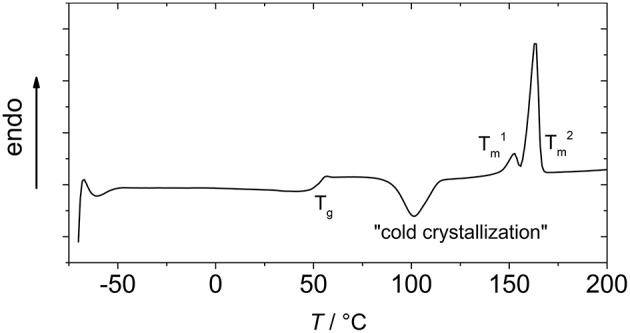
Representative DSC of PLLA synthesized by Fe(OAc)_2_ catalysis (with *N*-ethylacetamide as catalytic adjunct and at 105°C, column 6 of Table 1).

In Figure 4, a representative DSC curve of the PLLAs synthesized in this work is displayed. A T_g_, a cold crystallization, and two T_m_s were observed as thermal transitions in all samples. The glass transition was at 52 ± 1°C for all samples. This is well in the range of the reported T_g_ values for PLLA with an M_n_ ~15 kDa (Pan et al., 2007; Baker et al., 2008), though generally for PLLA, somewhat higher values are stated. It cannot totally be excluded that the measured samples contained small residues of lower molecular weight compounds that act as softeners, such as monomers, water, or amide, however, in any case the amount was so low that it was not observed in the NMR spectra.

Cold crystallization was observed at ~100°C (Eling et al., 1982). In the reported second heating run, two melting transitions (${\text{T}}_{\text{m}}^{1}$ and ${\text{T}}_{\text{m}}^{2}$) were observed. This has in the literature been rationalized by two different mechanisms. On the one hand, the polylactide may contain two populations of lamellae, the one with the lower melting transition corresponding to small lamellae formed during secondary crystallization, while the higher T_m_ corresponds to lamellae formed in primary crystallization (Su et al., 2009). On the other hand, the second (lower) melting transition may occur in melt recrystallization (Yasuniwa et al., 2004). The corresponding model of melt-crystallizations describes melting of small crystals and re-crystallization as competitive processes in the heating, and as these processes are relatively slow, the detection and relative integral of the cold crystallization peak and the ${\text{T}}_{\text{m}}^{1}$ in the DSC experiments is dependent on the heating rate.

The relatively low melting transition temperatures, ${\text{T}}_{\text{m}}^{1}$ and ${\text{T}}_{\text{m}}^{2}$, indicated a small crystallite size, which is in accordance with the observation that decreasing PLLA molecular weight correlates with decreasing melting transition temperatures when no curing is performed (He et al., 2007), and are furthermore dependent on the heating rate, as at lower heating rates more re-crystallization could take place. The increasing enthalpy of melting with decreasing temperature of the reaction gave evidence for an inverse correlation of crystallinity of PLLA and synthesis temperature, as long as the temperature was high enough to give high conversions and M_n_. When taking the literature value for Δ${\text{H}}_{\text{m}}^{∙}$ of 93 J·g^−1^ for PLLA (Fischer et al., 1973), the degrees of crystallinity (taking Δ${\text{H}}_{\text{m}}^{1}$ and Δ${\text{H}}_{\text{m}}^{2}$ into account) were between 30% and 54%.

Addition of *N*-ethylacetamide resulted in a more active catalyst system compared with *N*-methylbenzamide, as can be deducted from the more effective polymerization at 105°C when the former NPCA was used. As in the experiments reported in Table 1, 10 wt% of the respective NPCA was added and the molar masses of the two used NPCA were different, such difference in catalytic activity might have been connected with the different molar amounts used in the reactions (17 mol% vs. 11 mol%). In an additional set of experiments, therefore, the amount of *N*-methylbenzamide was increased to 31 mol%, while *N*-ethylacetamide was used in 11 mol% to study the influence of NPCA content (Table 2).

**Table 2:** Result of the polymerization reactions at 105°C after 4 h, with a catalyst/monomer ratio of 1:160, and under variation of NPCA type and amount.

	***N*-ethylacetamide**	***N*-methylbenzamide**
	**(11 mol%)**	**(31 mol%)**
Yield [mol%]	71	56
Conversion [mol%]	88	62
M_n, (NMR)_ [kDa]	14.1	9.6
M_n, (GPC)_ [kDa]	15	10.7
PDI	1.12	1.06
T_g_ [°*C*]^a^	51	47
Δ*C*_p_ [J·(*g*·*K*)^−1^]^a^	0.86	0.54
${\text{T}}_{\text{m}}^{1}$ [°*C*]^a^	152	148
$\Delta H_{\text{m}}^{1}$ [J·*g*^−1^]^a^	10	14
${\text{T}}_{\text{m}}^{2}$ [°*C*]^a^	162	158
$\Delta H_{\text{m}}^{2}$ [J·*g*^−1^]^a^	39	36
[α]^22^	−157	−158

*The theoretical M_n_ was 15 kDa*.

a *2^nd^ heating run. Two T_m_s were observed*.

*Precision of methods: NMR: ~10%, GPC: < 10%, DSC: Enthalpy ~10%, Temperature: 1 K*.

While the amount of NPCA did have an influence on the catalytic ROP at 105°C as the lowest investigated reaction temperature, *N*-ethylacetamide as NPCA resulted in more effective polymerization compared to N-methylbenzamide, even if the latter was used in higher quantities. The electronic properties of the two chosen NPCAs, in regard of their abilities to coordinate to Fe(II) while facilitating the coordination-insertion mechanism of the ROP, is likely differing. In fact, the rate of hydrolysis of aliphatic amides is faster than that of benzamides (Chapman, 1989), suggesting a higher polarity of the C = O bond in the acetamide. This would indicate a higher electron density at the carbonyl oxygen in the acetamide than in the benzamide, which may support coordination to Fe(II). The lower activity shown in the case of *N*-methylbenzamide compared to *N*-ethylacetamide could also be related to the steric hindrance in the first case.

**Figure 5:**
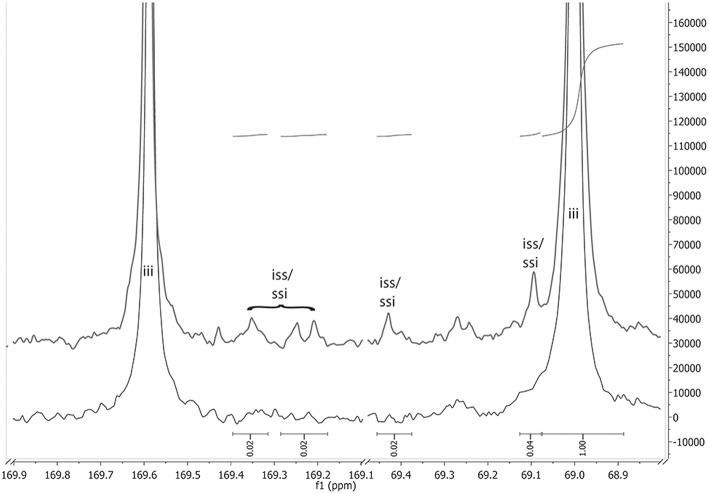
A comparison between the ^13^C-NMR expanded spectra of racemization free PLLA (bottom spectrum, reaction conditions: NEAA as NPCA, 105 °C; result column 6 in Table 1) and PLLA with small degree of racemization (top spectrum, reaction conditions: NEAA as NPCA, 165 °C; result column 3 in Table 1). The indicated integrals are referring to the top spectrum.

A critical point for the polymerization of optically active monomers is whether the chiral information is retained during the polymerization or is (partially) lost because of racemization. Increased temperatures during the polymerization are known to increase racemization (Ehsani et al., 2014), so that lowering the reaction temperature is beneficial. In order to detect racemization, the optical rotation of the synthesized polymers was measured. The values ([α]^22^ = −146° to −159°) are in good agreement with the literature (Yui et al., 1990; Pavlov et al., 2017). While an [α]^22^ ~ −146° might already indicate racemization to a minor extent, such a value was only obtained for the reaction with *N*-ethylacetamide as NPCA at 165°C, while at all other studied conditions, especially at lower polymerization temperatures, values around −155° were observed. Furthermore, racemization can be observed in ^13^C NMR spectra (Figure 5). Here, the excerpt of carbonyl- and the methine-carbon region of the ^13^C-NMR of PLLA, synthesized with *N*-ethylacetamide at 165°C (upper spectrum) and 105°C (lower spectrum), is shown. The main peaks at 169.6 ppm (carbonyl-C) and 69.0 ppm (methine-C) are related to iii-tetrades in the chain. At small degrees of racemization in the chain, in addition to the iii tetrades, ssi and iss tetrades, are expected to occur in the spectra (compare also Supplemental Information Figure S1 and Table S2), which is indeed the case for the sample synthesized at 165°C. The related peaks occur at ~169.2–169.4 ppm in the carbonyl region (Kricheldorf et al., 2008), and at 69.1 and 69.4 ppm in the methine region (Kasperczyk, 1999). Integration shows about ~4% of the iii signal in the region's representative for iss/ssi tetrades. Therefore, at 165°C configurational inversion occurred at about 4% of the lactic acid units. Because of very small signal size (and hence bad signal/noise ratio) and partial signal overlap, the error of this value is estimated to be around 20%. Concluding from the NMR spectra, it can be stated that indications for racemization were observed only with *N*-ethylacetamide as NPCA at a reaction temperature of 165°C, which is in line with the results from the polarimetry. This shows that the chosen catalyst system can be used without racemization.

Testing the limits of the catalytic system, polymerizations were run at a catalyst:momoner ratio of 1:750 instead of 1:160 and at a temperature of 105°C. The monomer:initiator ratio was kept constant in all experiments containing initiator at 1:104. In this case, the speed of reaction decreased so that even after 24 h of reaction, only a conversion of 68 mol% was observed, and the M_n_ remained <10 kDa. The PDI increased to 2 in these experiments, putatively a result of the prolonged reaction time that may allow for more transesterification (Full data: see supporting Table S1).

It is known that initiators such as the 1,8-octandiol used in this study increase the rate of ROP for catalysts like Sn(Oct)_2_ by lowering the energy of activation of the ring-opening (Kricheldorf et al., 1995). However, also in the absence of a co-initiator, polymerization is often observed in ROP, as nucleophilic impurities such as water or alcohols present in the catalyst can substitute for the specifically added co-initiator. This was also the case for Fe(OAc)_2_ –catalyzed ROP of dilactide, in which longer reaction times of 24 h were required to reach nearly quantitative (99 mol%) conversion of L,L-dilactide to PLLA. Here, M_n_s of PLLA up to 23.6 kDa were reached. Prior drying of the catalyst at 125°C under high vacuum for 24 h did not change the outcome of the reaction (full data: see supporting Table S1).

ROP polymerization is typically performed in the melt as the rate of polymerization is higher than in solution (Katiyar and Nanavati, 2010). However, side reactions, such as transesterification that may disturb sequence specificity, are reduced in solution so that it was also of interest to see whether the Fe(OAc)_2_/NPCA system would be active in solution. To explore the possibility to use the catalytic system in solution, NMP was chosen as a solvent, as it can act as NPCA and no further addition of another compound is required. ROP in NMP solution at 90°C resulted in a conversion of 87 mol% after 24 h and a PDI of 2.1. Hence, while the ROP can be performed under these conditions, the reaction is not as well controlled as in the melt.

**Figure 6:**
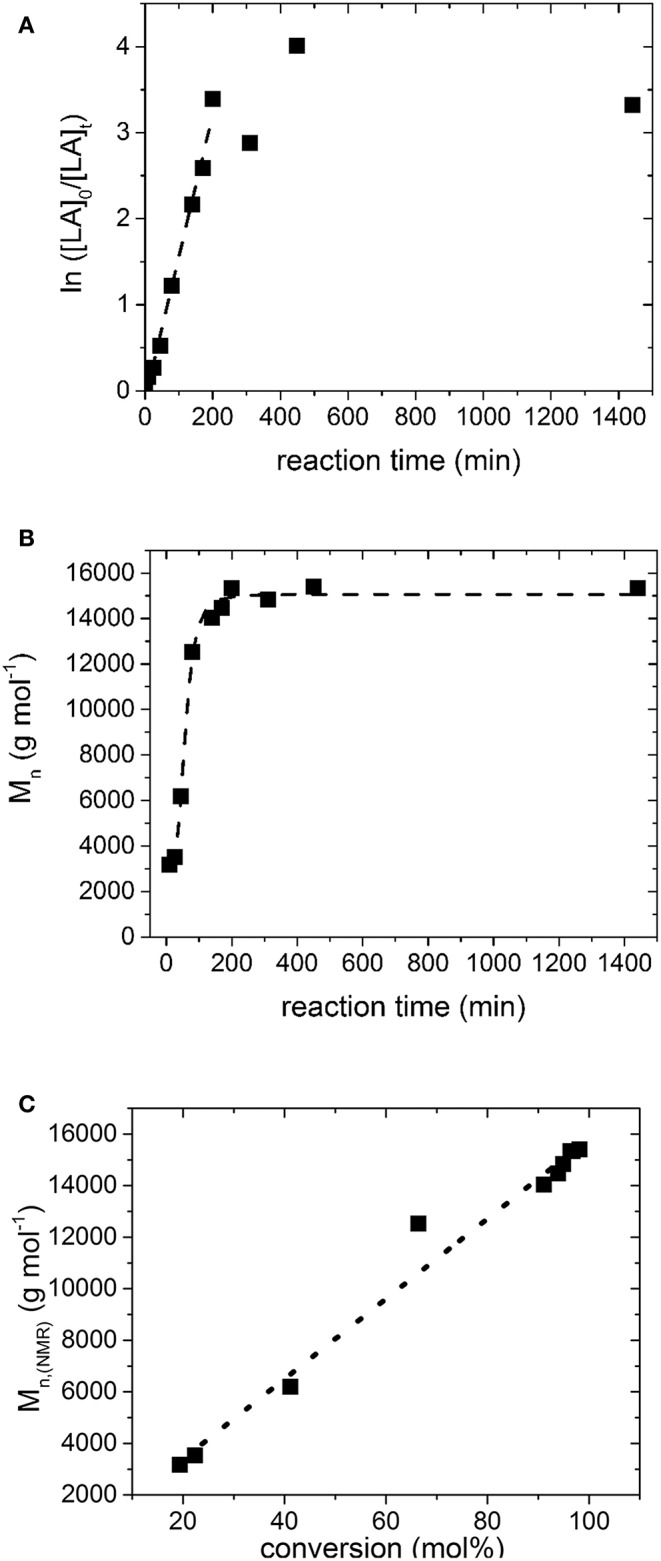
**(A)** Semilogarithmic plot of monomer conversion ratio vs. reaction time during the ROP of LLA using 1,8-Octanediol as initiator and Fe(OAc)_2_ as a catalyst in presence of *N*-ethylacetamide at 105°C; the linear regression has a Pearson R value of 0.99, **(B)** Plot of M_n_ vs. reaction time for the same experiment; the line was added as guide to the eye. **(C)** Plot of M_n_ vs. the conversion; the Pearson R value of the linear regression is 0.99. The data points were determined from the ^1^H NMR spectra and have an error of ~ 10%.

The kinetic of the ROP of L,L-dilactide with Fe(OAc)_2_ (catalyst:monomer = 1:160 mol), 1,8-octandiol as initiator (initiator:monomer = 1:104 mol) and 10 wt.% *N*-ethylacetamide (compared to the monomer), is depicted in Figure 6. >90% conversion was already reached after 2 h of reaction time. The observed linear dependence of conversion and reaction time in the semilogarythmic depiction (6A) and M_n_ and reaction time (6C) indicates a quasi-living chain growth mechanism up to high degrees of conversion (~95%), which is reflected furthermore by the low PDI of the synthesized polymers (see Table 1), and which has not been reached, with tin alkoxides, for example (Aubrecht et al., 2002).

**Figure 7:**
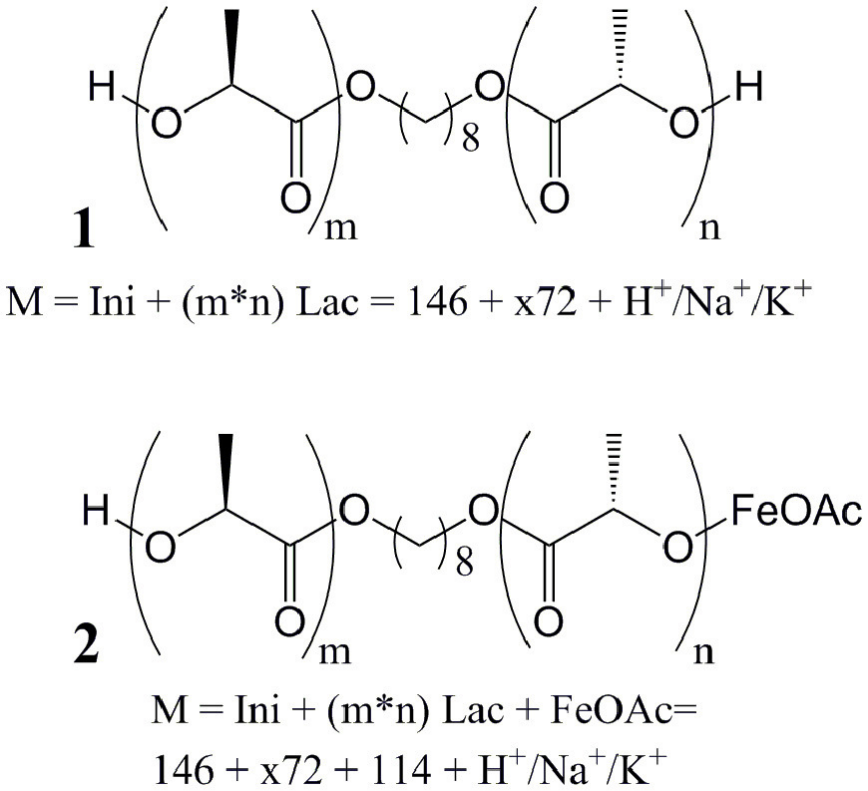
Structure of PLLA species found in the MALDI studies.

**Figure 8:**
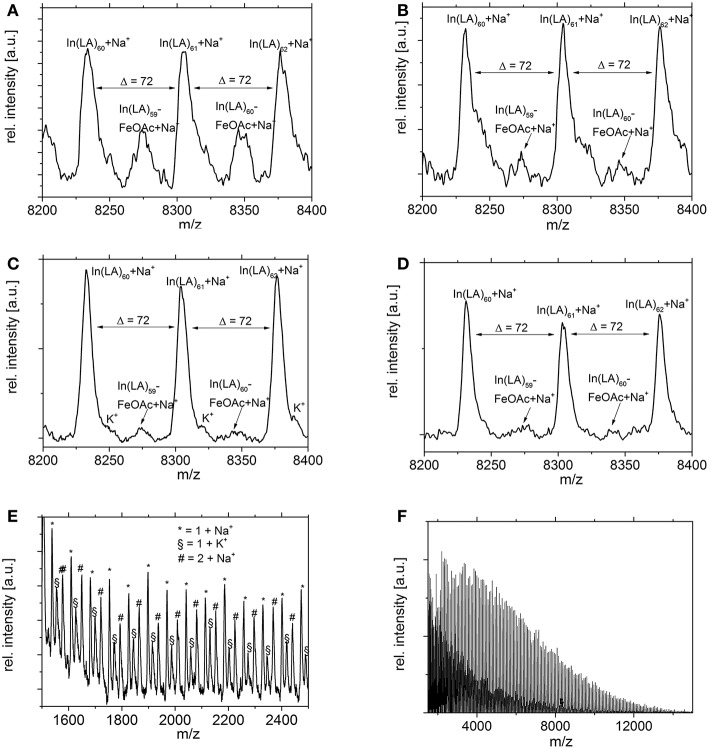
Excerpt of MALDI spectra of PLLA synthesized with *N*-Ethylacetamide as NPCA at **(A)** 165°C, **(B)** 145°C, **(C)** 125°C, **(D)** 105°C. The regular structure with a Δ = 72 m/z indicates transesterification. **(E,F)** Same conditions as D, different excerpt. At lower masses, the intensities of the Na^+^ and the K^+^-ions changes, but no other species are observed.

**Figure 9:**
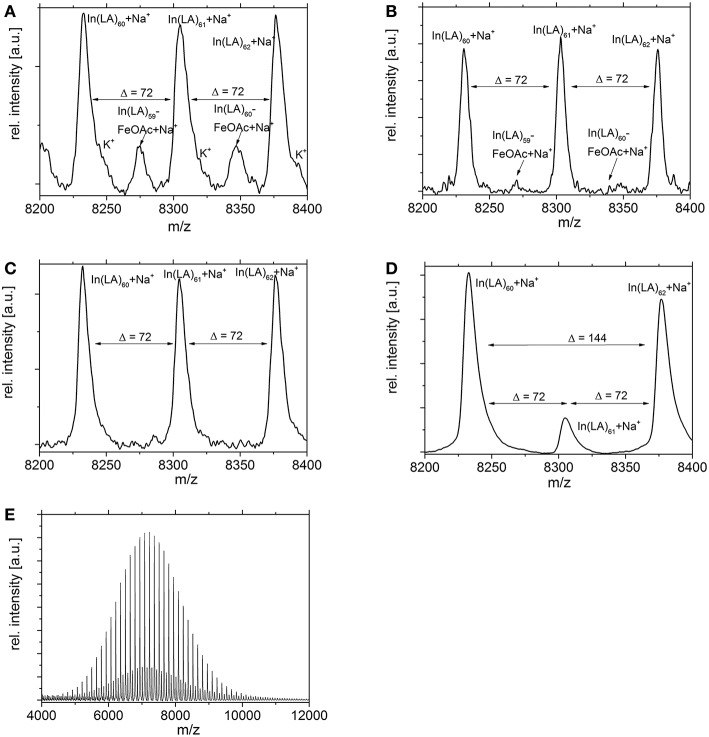
Excerpts of MALDI spectra of PLLA synthesized with *N*-Methylbenzamide as NPCA at **(A)** 165°C, **(B)** 145°C, **(C)** 125°C, or **(D)** 105°C. The regular structure with a Δ = 72 m/z indicates transesterification, which was observed only to a minor extend at 105°C **(D)**. **(E)** Same conditions as D, full spectrum.

MALDI studies (Figures 7–9) were conducted to investigate which species are present in the product in order to see whether side reactions such as cyclization and transesterification occurred, or if a noticeable fraction of chains were initiated by other nucleophiles other than 1,8-octanediol. In Figure 7, the species found in the MALDI are depicted. These are the targeted structure 1 as well as species 2, in which on one terminus, a FeOAc group is covalently attached. The direct observation of catalyst bound to the growing chain has been described before for Sn(Oct)_2_-catalyzed ROP (Kowalski et al., 2000), and is taken as evidence for the mechanism depicted in Figure 1. The structures of species potentially formed in side reactions, such as cyclic structures or polymers initiated by water, are depicted in Supplemental Information Figure S2, but none of these were found in the MALDI spectra. Spectra and excerpts of spectra are shown in Figure 8 (NEAA as NPCA) and Figure 9 (NMBA as NPCA). Figures 8A–D) shows the same region of the spectra for polymers synthesized at 165°C (Figure 8A), 145°C (Figure 8B), 125°C (Figure 8C), and 105 °C (Figure 8D), with the same species occurring in all spectra, while in Figures 9A–D, the spectra of polymers were synthesized at the same temperature in the presence of *N*-methylbenzamide as NPCA are depicted. Of the potentially formed species, only species 1 and 2 were observed (Figures 8, 9). The attachment of one FeOAc group at the chain end (species 2), present in most spectra, shows that the polymerization is well-controlled and that the predominant mechanism of ROP by Fe(OAc)_2_ is the mechanism depicted in Figure 1, and is analogous to the mechanism of Sn(Oct)_2_ catalyzed polymerization of lactones. The regular 72 m/z difference between ions showed that transesterification took place affecting the ester bond in a pure lactid diad. While transesterification in (co)polyesters based on dilactids has been observed for various catalysts, such as Sn(oct)_2_ (Kowalski et al., 2005), DBU (Meyer et al., 2010) and a Fe(II)-based ROP catalyst (Keuchguerian et al., 2015), the extent is largely differing depending on the reaction conditions, especially the temperature and some transesterification catalysts do not promote the transesterification of the ester bond of the lactide unit (Lendlein et al., 2000). In most of the conducted syntheses, the area of the peaks representing species containing an even number of lactide units was roughly equal to peaks representing species with an odd number of lactide units. However, when *N*-Methylbenzamide was used as NPCA and the reaction was conducted at 105°C, a much lower degree of transesterification was observed compared to the other cases. This coincides with a lower degree of monomer conversion at the studied time points and may suggest that for the catalyst system studied here, transesterification plays only a role at high conversions. The transesterification was not necessarily associated with a high PDI (compare Tables 1, 2) or with lowering of M_n_ with reaction time, which is otherwise typically observed. A potential rational for this observation may be that the coordination of ester groups is not totally random, but may occur preferentially at the sterically more easily accessible end groups of the polymers, which, after transesterification, would only lead to small changes in the PDI. The mass spectra did not display species 8–10 related to ring formation (Figure S2). No end groups with attached acetate or benzoate were found (species 11–13), therefore, it could be shown that the acyl part of the NPCA in fact was not transferred to the growing chain. Figure 8E shows another region of the same spectrum as in Figure 8D, demonstrating exemplarily that no additional species were observed in other regions of the spectra. Figures 8F, 9E depict the full spectrum. Also, only the presence of the above-mentioned species can be observed here. 8F may give the impression that there is an additional species present at lower masses, however, this is only a change of relative intensity of the M+Na^+^ ions compared to the M+K^+^ ions.

## Conclusions

It was shown that the addition of NPCAs to Fe(OAc)_2_ enabled the ROP of lactide at temperatures down to 105°C. Such low polymerization temperatures were associated with low polydispersities and no racemization of the polymers. The catalyst system is easily available through the mixing of commercially available compounds *in situ*, which supports a widespread use. Furthermore, Fe-based compounds putatively have a lower toxicity than Sn-based compounds. The lower temperatures required for the synthesis compared to a Sn(Oct)_2_-catalyzed ROP means that less energy is consumed, which is economically beneficial for larger scale reactions. Therefore, the introduced system is of interest to the synthetic polymer chemist. In further studies, the exact structure of the active catalyst would need to be elucidated. As other catalysts employed for the ROP of lactide are also catalyzing the ROP of other lactones, such as ε-caprolacton, glycolide, or *p*-dioxanone, and as copolymers from these monomers are generally used rather than the homopolymers, the introduced catalytic systems will be tested for the homo- and copolymerization of such lactones in the future. Furthermore, the influence of the NPCA will have to be evaluated by systematic variation of their properties and structures.

## Data Availability

The raw data supporting the conclusions of this manuscript will be made available by the authors, without undue reservation, to any qualified researcher.

## Author Contributions

ATN contributed conception and design of the study. TN performed the experiments and contributed selected experiments as well as experimental setup. TN, AL, and ATN analyzed and interpreted the data. TN wrote the first draft of the manuscript. TN, AL, and ATN wrote sections of the manuscript. ATN was responsible for writing the final version. All authors contributed to manuscript revision, read and approved the submitted version.

### Conflict of Interest Statement

The authors declare that the research was conducted in the absence of any commercial or financial relationships that could be construed as a potential conflict of interest.
