# Supplementary material for: Amides as Non-polymerizable Catalytic Adjuncts Enable the Ring-Opening Polymerization of Lactide With Ferrous Acetate Under Mild Conditions
Source: Front Chem. 2019 May 16;7:346. doi: 10.3389/fchem.2019.00346 (PMC6535497; doi:10.3389/fchem.2019.00346)
Supplement: Supplementary file 1 [file Presentation_1.pdf]

Supplementary Material

Amides as non-polymerizable catalytic adjuncts enable the ring-opening polymerization of lactones with ferrous acetate under mild conditions

By Toufik Naolou, Andreas Lendlein and Axel T. Neffe

Table S1. Result of the polymerization reactions at 105 °C under variation of catalyst amount and reaction time, with/without initiator, or when performing the reaction in NMP as solvent. The theoretical M_n was 15 kDa.

	Low catalyst[a]			No initiator				NMP
N-ethylacetamide [mol%]	17	4	17	17	17	17	11	-
Time [h]	4	4	24	4	8	24	24	24
Yield [mol%]	38	30	45	48	30	88	71	62
Conversion [mol%]	48	35	68	68	88	99	99	87
$M_{n,(NMR)}$ [kDa]	8.3	6.4	9.4	*	*	*	*	17.1
$M_{n,(GPC)}$ [kDa]	5.5	5	3.7	17.0	10.5	23.6	12.5	4.3
PDI	1.4	1.2	2	1.4	3.2	1.8	2.2	2.1
T_g [°C][b]	44	43	50	57	57	52	56	50
ΔC_p [J·(g·K)$^{-1}$][b]	0.33	0.46	0.43	0.63	0.54	0.55	0.74	0.35
T_m^1 [°C][b]	142	132	143	170	166	165	160	149
ΔH_m^1 [J·g^{-1}][b]	19	14	19	44	38	48	18	12
T_m^2 [°C][b]	151	142[c]	151	-	-	-	-	159
ΔH_m^2 [J·g^{-1}][b]	24	30	21	-	-	-	-	29
$[\alpha]^{22}$	-149	-156	-153	-159	-155	-159	-154	-152

a: Catalyst/monomer ratio: 1:750. In all other experiments, this ratio was 1:160. The monomer:initiator ratio was kept constant in all experiments (other than the experiments with no initiator) at 1:104. * In the experiments without initiator, the M_n determined by ^{1}H NMR was in all cases >> 40 kDa. The error in NMR-based determination of M_n by comparison of the integrals related to protons of internal repeating units with the ones of end groups gets very large in these cases and is therefore not reliable. b: 2nd heating run. c. Two peaks 141/144 °C. Precision of methods: NMR: ~10%, GPC: <10%, DSC: Enthalpy ~10%, Temperature: 1 K.

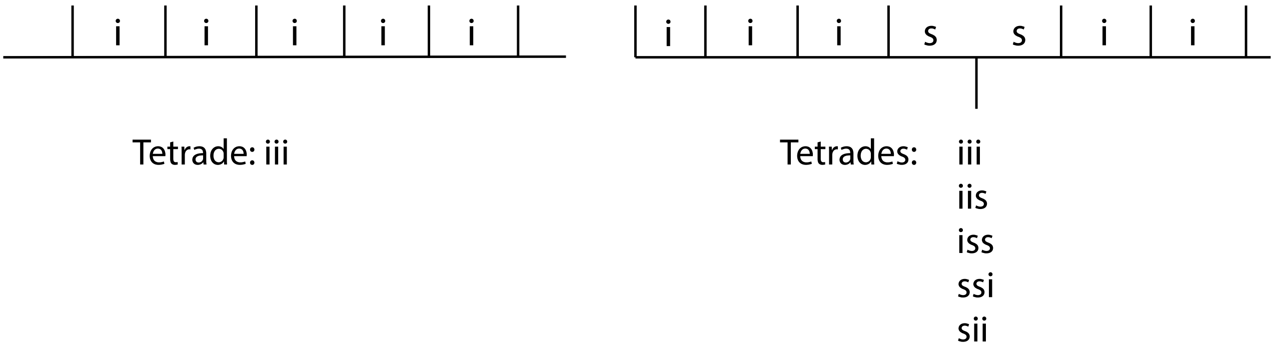

Figure S1. Tetrades potentially occurring in PLLA (left) and partial racemized PLLA (right)

Table S2. ^{13}C chemical shifts of the carbonyl group and the methine carbon in different tetrades according to Kricheldorf, H.R., Lomadze, N., Schwarz, G. (2008). Cyclic Polylactides by Imidazole-Catalyzed Polymerization of L-Lactide. *Macromolecules* 41, 7812-7816. and Kasperczyk, J.E. (1999). HETCOR NMR study of poly(rac-lactide) and poly(meso-lactide). *Polymer* 40, 5455–5458.

.

Group	Tetrade chemical shift	
	iii	iss, ssi*
Carbonyl	169.6	169.4-169.2
Methin	69.0	69.1, 69.4

* interestingly, for the tetrades iis and sii, no clear values could be found in literature (in addition to the above cited references, e.g. the publications Macromolecules 1999, 32, 963-973; Chem. Commun., 1998, 1913-1914; J. Macromol. Sci. A Pure Appl. Chem. 1993, 30:6-7, 441-448; and Biomacromolecules 2011, 12, 3299–3304. address the assignment of tacticity in polylactide). Typically, s-containing tetrades have lower chemical shifts in the carbonyl region than the pure iii, while in the methine region, higher chemical shifts for s-containing tetrades are observed than for iii.

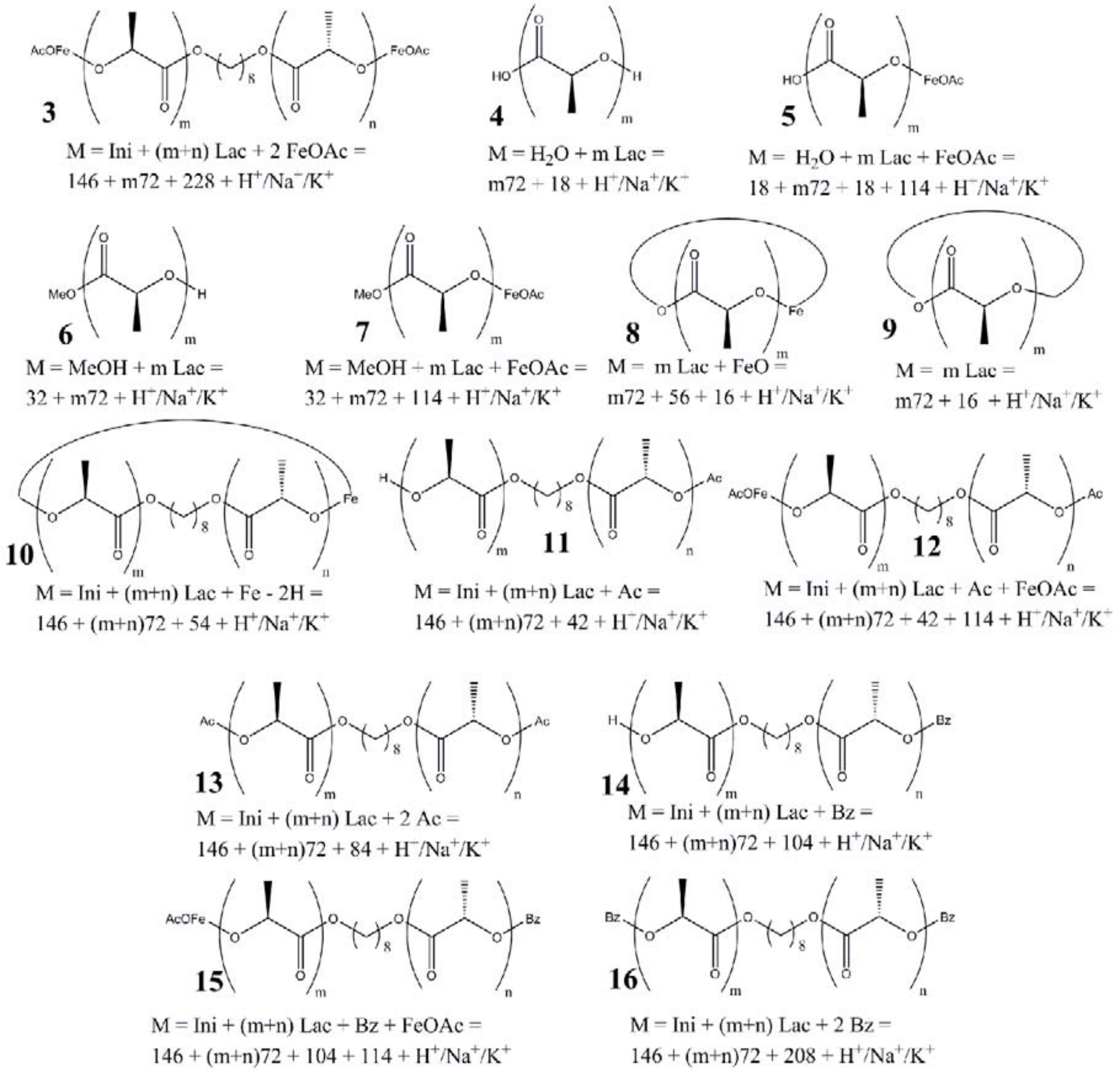

Figure S2. Structure of species potentially formed in side reactions of the polymerization, but not found in the MALDI studies. Species 3 bears two catalysts and would indicate a simultaneous growth on two sides. Species 4 and 5 would occur when residual water initiates chains, while species 6 and 7 may occur in case of initiation by water or one of the OAc ligands, and a subsequent ester formation in the methanol precipitation step. Species 8-10 are macrocycles, 8 and 10 including the bound catalyst. Species 11-13 or 14-16, respectively, may occur in case of transfer of the acyl moiety of the NPCA to the growing chain. This means that compounds 11-13 can only occur in synthesis under the addition of NEAA as source of the acetyl group, while species 14-16 would require the presence of NMBA as source of the benzyl group.
